# Osteohistological analysis reveals unusual bone tissue patterns in Temminck's ground pangolin (*Smutsia temminckii*)

**DOI:** 10.1098/rsos.261533

**Published:** 2026-10-28

**Authors:** Palesa Mamabolo, Francesca Parrini, Jennifer Botha

**Affiliations:** ^1^ School of Animal, Plant and Environmental Sciences, University of the Witwatersrand, Johannesburg, Gauteng, South Africa; ^2^ GENUS: DSTI-NRF Centre of Excellence in Palaeosciences, University of the Witwatersrand, Johannesburg, Gauteng, South Africa; ^3^ Evolutionary Studies Institute, University of the Witwatersrand, Johannesburg Faculty of Science, Johannesburg, Gauteng, South Africa

**Keywords:** bone histology, mammal bone microstructure, pangolin growth, growth patterns, compacted coarse cancellous bone 1

## Abstract

Temminck's ground pangolin is one of Africa's most threatened mammals, yet little is known about its skeletal biology. This study provides the first osteohistological analysis of its limb bones, documenting patterns of growth, remodelling and skeletal reinforcement. Limb bones exhibit extensive cortical remodelling, with substantial portions of the cortex being composed of compacted coarse cancellous bone, endosteal bone and secondary osteons, together with abundant resorption cavities. Growth marks occur in all elements, but the absence of outer circumferential lamellae and persistence of open vascular canals indicate continued growth and a lack of skeletal maturity despite the presence of up to four annual growth cycles. Extensive skeletal reinforcement occurs throughout the limbs, although elements employ distinct osteohistological patterns. Comparison with the aardvark reveals alternative pathways of skeletal reinforcement, with ground pangolins showing relatively greater secondary remodelling in the hind limb. Ground pangolins are further distinguished by abundant, incompletely infilled resorption cavities extending into the middle and outer cortex, suggesting an unusual pattern of intracortical remodelling. The exceptionally low basal metabolic rate of *S. temminckii* may contribute to these patterns, although this relationship remains to be tested. These findings provide new insights into the skeletal biology of this poorly studied mammal.

## 1. Introduction

Temminck's ground pangolin, *Smutsia temminckii*, is a solitary, primarily nocturnal, myrmecophagous, facultatively bipedal mammal distributed throughout southern and eastern Africa [1–7]. It can reach lengths of up to 140 cm and weigh as much as 12 kg [3]. The species is characterized by large, overlapping keratinous scales that cover the dorsal and lateral surfaces of the body, the limbs, forehead and both the dorsal and ventral surfaces of the tail [3].

Despite its broad geographic distribution, the ground pangolin is considered one of the most threatened mammals in Africa. The species faces numerous anthropogenic pressures, including accidental electrocution on electric fences and overexploitation for the traditional African medicine trade, which are currently regarded as the primary causes of mortality [1–3,6,8]. Additional threats include habitat loss associated with subsistence and crop agriculture, poaching for bushmeat consumption, road mortalities and capture in gin traps. Consequently, the ground pangolin is listed as Vulnerable on the IUCN Red List. Because populations are declining and many aspects of its biology remain poorly understood, any new information regarding the anatomy, physiology, growth and ecology of this species is of considerable conservation importance, providing the baseline needed to inform management, rehabilitation and monitoring.

Ground pangolins are burrow-associated mammals and are classified primarily as scratch diggers. Unlike many fossorial mammals, they do not excavate their own burrows but instead occupy and modify those originally constructed by other species, including aardvarks (*Orycteropus afer*), Cape porcupines (*Hystrix africaeaustralis*), springhares (*Pedetes capensis*) and warthogs (*Phacochoerus* spp.). Nevertheless, they possess several pronounced morphological adaptations for digging, largely related to foraging behaviour, as they actively excavate ant nests and termite mounds. These adaptations include a robust humerus, an elongated olecranon process of the ulna and three enlarged, powerful claws used to break open termitaria and modify burrow systems [3,9].

Previous studies on ground pangolins have focused predominantly on behaviour [3,10], diet [2,3,5,10], ecology [3,8,10], physiology [3,11], conservation [1,3,12] and reproduction [3,10]. However, no study has yet investigated the bone histology of this species. Osteohistology provides valuable insight into growth dynamics, growth rates, ontogeny and life-history strategies, while bone microanatomy can also reflect biomechanical loading and functional adaptations associated with specific lifestyles. Previous osteohistological studies of fossorial and digging mammals, including the Cape dune mole-rat (*Bathyergus suillus*), aardvark and nine-banded armadillo (*Dasypus novemcinctus*), have demonstrated that bone microstructure can reveal important information regarding locomotory behaviour, mechanical stress and adaptations to digging lifestyles [13–15].

This study therefore provides the first detailed description of the osteohistology and bone microanatomy of the ground pangolin. Specifically, it investigates growth patterns and growth rates, while also assessing the extent to which specialized digging adaptations influence limb bone microstructure. In doing so, the study contributes novel baseline biological data for one of Africa's most threatened mammals and expands current understanding of the relationship between digging behaviour and skeletal microstructure in mammals.

## 2. Material and methods

### 2.1. Material

The humerus, radius, ulna, femur, tibia and fibula from five specimens were sampled in this study. Two specimens (BP/4/1342 and BP/4/1343) were relatively complete, allowing all the limb bones to be sampled from each individual. Additional midshaft limb-bone material was sampled from three specimens: BP/4/1344 (forelimb), BP/4/1346 (radius, femur and tibia) and BP/4/134 (forelimb, femur and tibia). The material was donated by the African Pangolin Working Group. The material is housed in the Evolutionary Studies Institute (BP; formerly the Bernard Price Institute), University of the Witwatersrand, South Africa.

The absolute ontogenetic ages of the individuals are unknown. However, the partial preservation and unfused state of the limb bone epiphyses in BP/4/1342 and BP/4/1343 indicates that these specimens likely represent a subadult skeletal maturity. The remaining specimens (BP/4/1344, 1346, 1347), previously examined in a study on the thoracic limb osteology of *Smutsia temminckii* [7], weighed 7–8 kg and have fused epiphyses, indicating a subadult to adult skeletal maturity [7]. As fully grown ground pangolins can weigh as much as 12 kg [3], they had yet to reach maximum size.

### 2.2. Methods

All bones were pretreated prior to thin sectioning following the protocol outlined for the aardvark by [14]. Thin sections were then prepared at the Evolutionary Studies Institute, University of the Witwatersrand, using standard methods detailed in [16], and imaged using a Nikon Eclipse Ci POL polarizing microscope fitted with a DS-Fi3 digital camera, operated in NIS-Elements v. 4.6 (Nikon Corp.). Osteohistological terminology follows [17]. High-resolution images of the original sections have been reposited on MorphoSource [18].

### 2.3. Analyses

In addition to qualitative osteohistological descriptions, quantitative data on bone tissue proportions and microanatomical parameters were collected. A midshaft transverse-section micrograph of each bone was analysed in the GNU Image Manipulation Program (GIMP; v. 2.10.38) to quantify the proportional areas of endosteal bone, primary bone, compacted coarse cancellous bone (CCCB), secondary osteons and Sharpey's fibres (as indicators of muscle attachment) within the section. The area of each feature was measured and expressed as a percentage of the total bone wall area. The same procedure was applied to limb bones of the aardvark to enable quantitative comparison of tissue proportions and the relative prominence of Sharpey's fibres.

Binary images (bone = black; void space = white) generated from the same micrographs were uploaded to Bone Profiler (v. 4.5.8.56) to calculate global compactness (Cg) and the cortical thickness (CDI; cortico-diaphyseal index) [19,20]. Higher Cg values indicate a more compact bone wall and higher CDI values, a thicker cortex. These values were also calculated for the aardvark and compared to the ground pangolin.

## 3. Results

### 3.1. Humerus

Four humeri were examined in this study. The humerus is the longest element of the thoracic limb [7]. Transverse sections are generally triangular (figure 1a) because they include the humeral crest, with the exception of BP/4/1344, which was sampled from a more distal region. Overall, the cortex of the humeri is not particularly thick having a CDI of 0.45 (figure 1a) and the medullary cavity is relatively large. The overall cortical compactness is relatively high (Cg = 0.72). Only small patches of primary bone are preserved in the inner cortex, as this region has been extensively remodelled. Where present, the primary inner cortex consists of a woven matrix containing abundant, large, globular, disorganized osteocyte lacunae (figure 1b). This tissue is associated with longitudinally oriented primary osteons, indicating a rapidly deposited woven–parallel complex (WPC). In the mid- and outer cortex, osteocyte lacunae become more organized and locally arranged into parallel rows, consistent with a transition to a more parallel-fibred matrix (figure 1b). This change is accompanied by reduced vascularization, with primary osteons becoming sparse and vascular canals dominated by small, longitudinally oriented simple canals. Endosteal bone lines most of the medullary cavity, forming locally thickened regions (figure 1c,d). On the cranial face, adjacent to the humeral crest, the endosteal bone forms cancellous trabeculae that increasingly infill the medullary cavity towards the proximal portion of the humerus. Where endosteal bone is absent, the medullary margins are composed of CCCB that extends into the inner cortex and locally into the mid-cortex (figure 1e–h). Approximately 20% of the cortex comprises CCCB (table 1).

**Figure 1.**
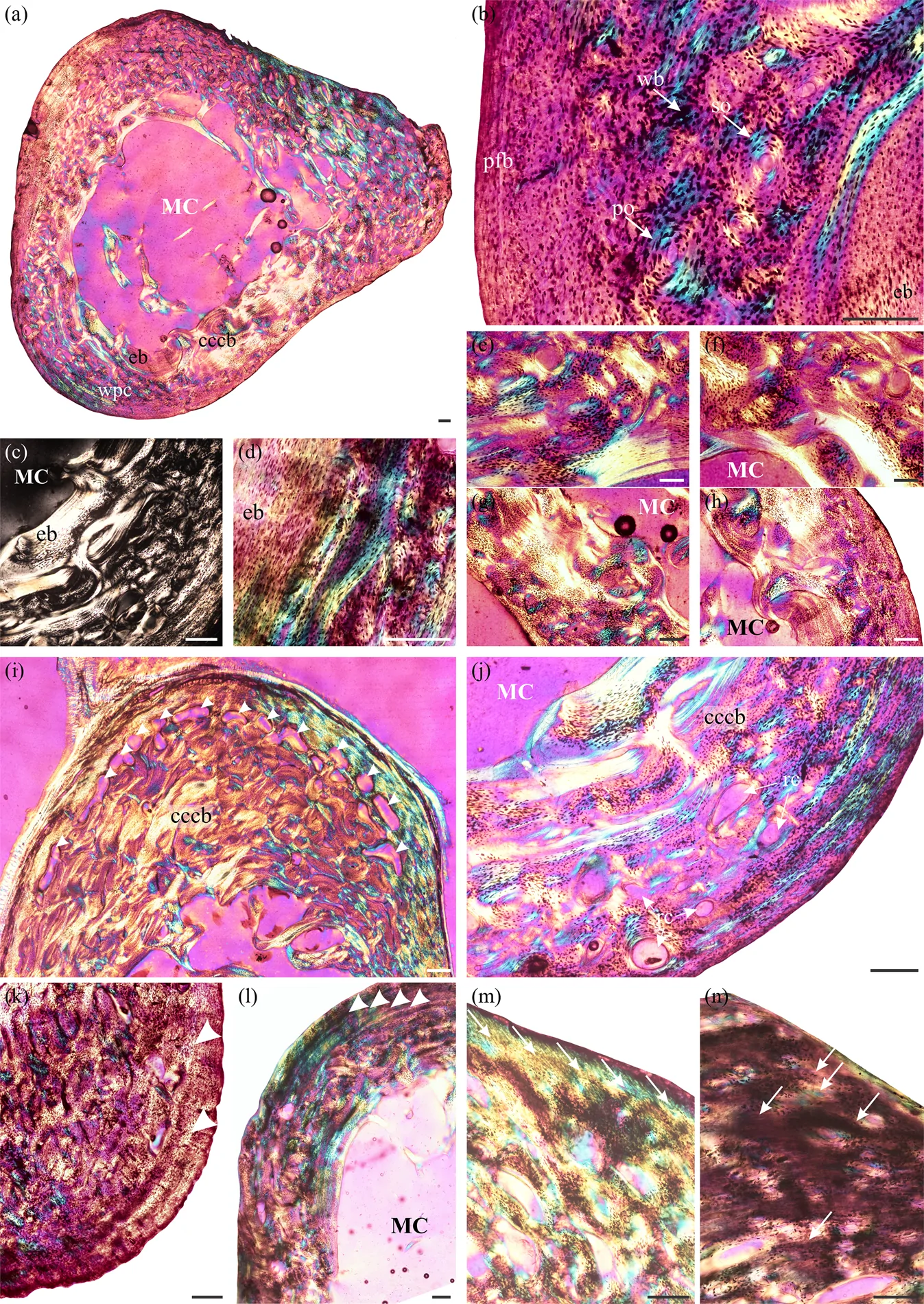
Humeral osteohistology of *Smutsia temminckii*. (a) Triangular cross-sectional view of the midshaft of BP/4/1342. (b) Woven–parallel complex comprising woven bone and primary osteons in BP/4/1342. (c,d) Thick inner layer of endosteal bone. (e–h) Various sections of the inner cortex of BP/4/1342 showing compacted coarse cancellous bone. (i) BP/4/1344 showing compacted coarse cancellous bone and a row of large resorption cavities in the outer cortex. (j) BP/4/1342 showing compacted coarse cancellous bone and large resorption cavities in the mid and outer cortex. (k) BP/4/1343 showing two annuli and an uneven surface. (l) BP/4/1347 showing four annuli and LAGs. (m,n) BP/4/1343 and BP/4/1347 showing Sharpey's fibres indicating muscle insertions (arrows). Arrowheads indicate growth marks. Abbreviations: CCCB, compacted coarse cancellous bone; EB, endosteal bone; MC, medullary cavity; PFB, parallel-fibred bone; PO, primary osteon; RC, resorption cavity; SO, secondary osteon; WB, woven bone. Scale bars, 250 µm.

**Table 1.** Quantification of bone tissue type in limb bones of the ground pangolin (*Smutsia temminckii*) BP/4/1342 and the aardvark (*Orycteropus afer*) measured as a percentage (see figure 7). CCCB, compacted coarse cancellous bone; EB, endosteal bone; PB, primary bone; Shf, Sharpey's fibres; SO, secondary osteons.

species	element	EB %	CCCB %	PB %	SO %	Shf %
*Smutsia temminckii*	humerus	18	20.1	24.6	35.6	1.7
	radius	14	29.8	28.4	24.4	3.4
	ulna	3.1	12.6	53	31.3	0
	femur	15	20.9	36.2	27.9	0
	tibia	13	7.1	31.9	48	0
	fibula	8	19.1	23.2	49.7	0
*Orycteropus afer*	humerus	12.2	58.2	3.4	26	0.2
	radius	4.8	58.6	22	13	1.6
	ulna	0	46.6	22.8	28.9	1.7
	femur	13.8	34.2	22.1	29.5	0.4
	tibia	1.5	39.4	26	33.1	0
	fibula	12.8	26.4	22.7	38.1	0

Secondary osteons are concentrated primarily in the mid-cortex but extend into the outer cortex along the humeral crest, where they are commonly associated with Sharpey's fibres. Secondary remodelling is extensive and includes both fully developed secondary osteons and resorption cavities that remain partially or entirely unfilled. Many of these cavities and partially infilled secondary osteons occur in the mid- to outer cortex, either forming rows suggestive of a remodelling front (e.g. figure 1i) or occurring more randomly among areas of remodelled and primary bone (figure 1j). Growth marks, expressed as annuli (temporary decreases in growth) or lines of arrested growth (LAGs; temporary cessations), occur in the mid- and outer cortex, with two to three in most individuals and four in BP/4/1347 (figure 1k,l). In BP/4/1342 and BP/4/1343, the subperiosteal region is locally wavy and contains open vascular canals, indicating continued growth at the time of death (figure 1k). This is further supported by the absence of clear outer circumferential lamellae or an OCL (also known as an EFS or External Fundamental System) suggesting that these animals were still actively growing when they died. Prominent Sharpey's fibres are present in nearly all individuals along the humeral crest (figure 1m,n).

### 3.2. Radius

Five radii were available for examination. The radial shaft comprises a rounded proximal ‘neck’ that extends for approximately one-third of the element's length before transitioning into the body, which is flattened craniocaudally. Midshaft transverse sections taken through the neck region are circular and show a moderately thick cortex with a CDI of 0.51 (e.g. BP/4/1342, 1344; figure 2a). The cortical compactness is relatively high with a Cg of 0.75. In most specimens, the inner primary cortex has been extensively remodelled. However, BP/4/1347 preserves a mid- to outer cortical region of primary compact bone in which osteocyte lacunae range from well-organized to more haphazardly arranged (figure 2b,c). In a few patches near the subperiosteal surface, osteocyte lacunae form parallel, wave-like rows indicative of parallel-fibred bone (PFB), whereas other areas retain clear woven bone. This tissue is moderately vascularized, with predominantly longitudinal simple vascular canals and a small number of poorly developed primary osteons. Despite this localized preservation of primary tissue, the compact cortex of the radii is dominated by variably thick layers of endosteal bone and CCCB (approx. 30% of the cortex; table 1), together with abundant secondary osteons (figure 2d,e). In some regions, the outer cortex remains unremodelled and consists of poorly vascularized PFB. Growth marks interrupt the cortex and are not restricted to the outermost periosteal region. For example, in BP/4/1347 both a mid-cortical and an outer LAG are visible (figure 2b,c). Large resorption cavities are common throughout the mid-cortical CCCB (figure 2d–g). Osteohistological differences are also apparent between sections sampled from the neck versus the body of the radius. Neck-region sections typically retain an open medullary cavity. By contrast, sections taken from the flattened body show partial infilling by endosteal trabeculae (figure 2d,g), and the endosteal margin is more extensively resorbed, with large resorption cavities extending into the inner cortex. These large inner-cortical cavities tend to be absent closer to the neck, where the endosteal/CCCB region remains relatively compact, and resorption cavities occur mainly in the mid- and outer cortex (figure 2h). An OCL was not observed in any of the radii. Sharpey's fibres (figure 2i–k) are observed in BP/4/1342 near the proximal neck region, consistent with the biceps brachii insertion near the radial tuberosity, and along the medial and lateral shaft margins in BP/4/1343 and BP/4/1346, probably reflecting strong attachment of the pronator musculature medially and the interosseous membrane laterally.

**Figure 2.**
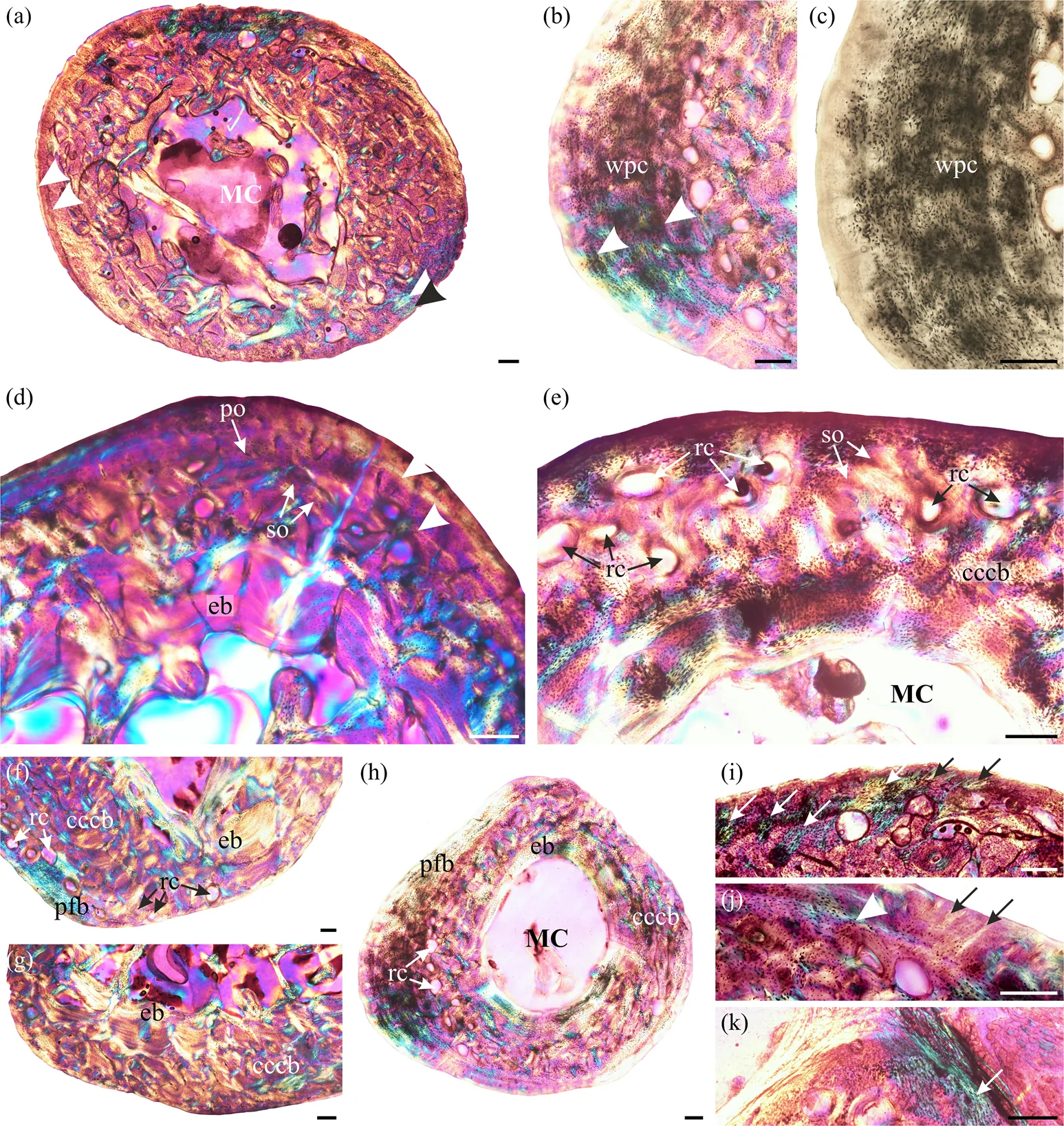
Radial osteohistology of *Smutsia temminckii*. (a) Complete transverse section of BP/4/1342 showing two annuli. (b) BP/4/1347 showing a woven–parallel complex and two annuli in cross-polarized light. (c) BP/4/1347 showing a woven–parallel complex in normal light. (d) BP/4/1343 showing a thick inner layer of endosteal bone, a mostly remodelled cortex with secondary osteons. (e) BP/4/1342 showing compacted coarse cancellous bone, large resorption cavities and secondary osteons. (f) BP/4/1346 showing a thick inner region of endosteal bone, an inner region of compacted coarse cancellous bone and resorption cavities, and a small outer region of parallel-fibred bone. (g) BP/4/1346 showing a thick layer of endosteal bone and a thick region of compacted coarse cancellous bone. (h) Complete transverse section of BP/4/1347 showing thick inner regions of endosteal bone, compacted coarse cancellous bone and large resorption cavities and an outer region of parallel-fibred bone. (i) BP/4/1342, (j) BP/4/1343, (k) BP/4/1344 showing Sharpey's fibres indicating muscle insertions (arrows). Arrowheads indicate annuli. Abbreviations: CCCB, compacted coarse cancellous bone; EB, endosteal bone; MC, medullary cavity; PFB, parallel-fibred bone; PO, primary osteon; SO, secondary osteon; WPC, woven–parallel complex. Scale bars, 250 µm.

### 3.3. Ulna

Four ulnae were analysed. The ulna is a craniocaudally flattened bone. More proximal midshaft sections closer to the lateral process reveal a thick compact cortex (CDI = 0.92) and relatively small open medullary cavity (figure 3a). Sections from the rest of the midshaft show a predominance of CCCB (figure 3b). The primary bone tissue comprises a well vascularized WPC with longitudinally oriented primary osteons (figure 3a,c). Sections show one to two growth marks either as annuli of lamellar bone or LAGs (figure 3a,b). Outer primary regions are composed of poorly vascularized PFB (figure 3d). Numerous large resorption cavities or partially infilled secondary osteons are distributed throughout the mid-cortex (figure 3b), with some forming rows at the boundary between the CCCB and primary bone in the outer cortex (figure 3e,f). Outer Circumferential Lamellae were not observed. Sharpey's fibres are visible in some sections (figure 3g,h), possibly marking the attachment of extensor and supinator musculature (potentially including abductor pollicis longus and related deep extensors).

**Figure 3.**
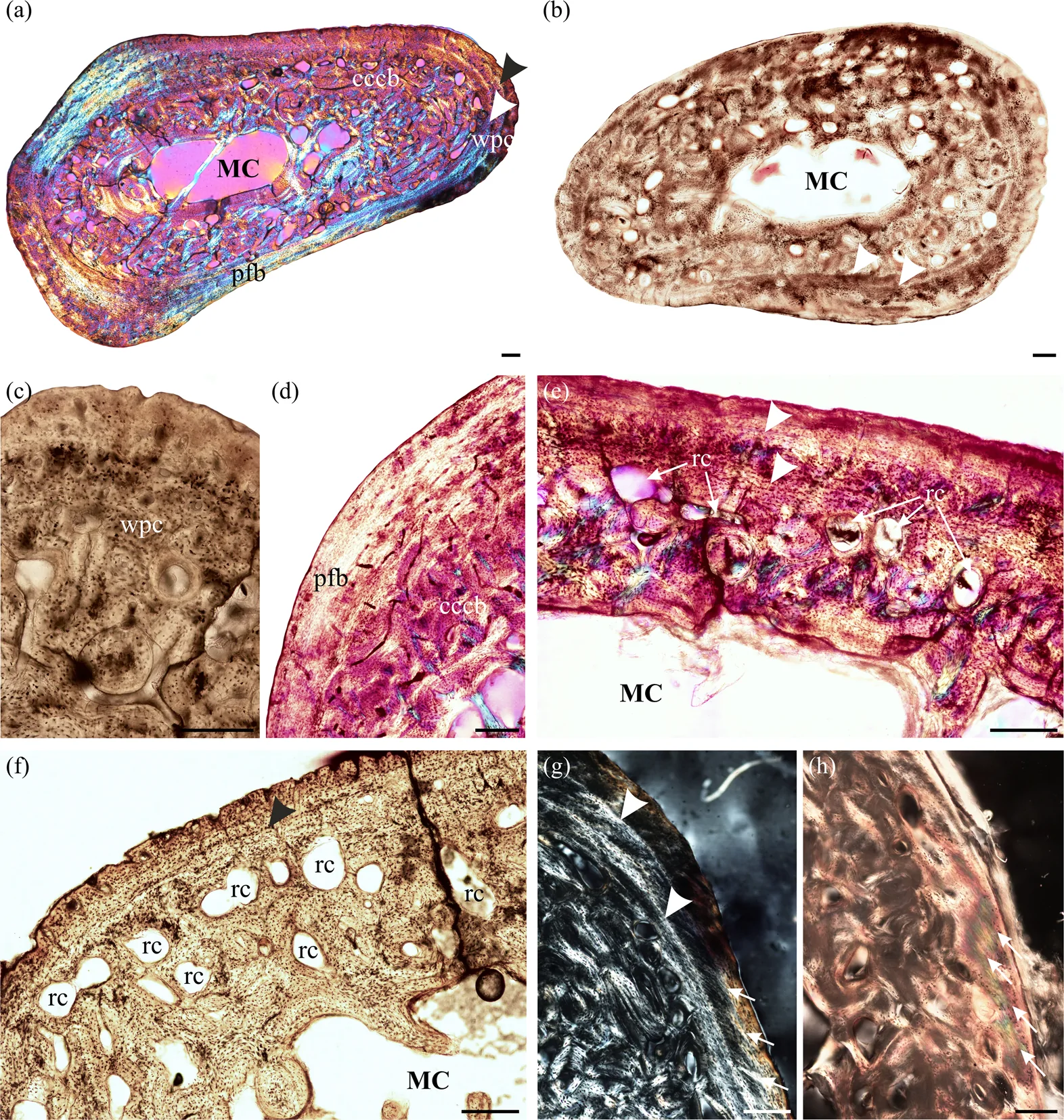
Osteohistology of the ulna of *Smutsia temminckii*. (a) Whole cross-section of BP/4/1343 showing a relatively thick cortex and a small medullary cavity, and two growth marks. (b) Complete transverse section of BP/4/1347 showing compacted coarse cancellous bone and numerous large resorption cavities, and two annuli. (c) BP/4/1342 showing a woven–parallel complex. (d) BP/4/1344 showing an outer region of parallel-fibred bone. (e) BP/4/1342 and (f) BP/4/1343 showing compacted coarse cancellous bone and mid-cortical resorption cavities. (g) BP/4/1343 and (h) BP/4/1347 showing Sharpey's fibres (arrows) indicating muscle insertions. Arrowheads indicate growth marks. Abbreviations: CCCB, compacted coarse cancellous bone; MC, medullary cavity; PFB, parallel-fibred bone; RC, resorption cavity; WPC, woven–parallel complex. Scale bars, 250 µm.

### 3.4. Femur

Four femora were available for study. The hind limb is proportionally longer than the forelimb, with the femur exceeding the humerus in length. Each femur comprises a long, straight midshaft. Midshaft transverse sections reveal a relatively thin cortex (CDI = 0.47) in which much of the primary tissue has been remodelled. However, portions of the mid-cortex and parts of the outer cortex retain unremodelled primary bone (figure 4a–d). This primary tissue consists of a well-vascularized woven–parallel complex (figure 4b) that transitions to PFB towards the subperiosteal surface (figure 4c). Vascularization, expressed mainly as longitudinally oriented primary osteons, decreases in the outer cortex and grades into simple vascular canals within the outer PFB. Growth marks occur in both the mid- and outer cortex, with up to four visible in specimen BP/4/1342 (figure 4d). No clear OCL was observed in any femur. Endosteal bone lines the medullary cavity and locally becomes thickened in some of the sections (figure 4e,f). At the midshaft, the medullary cavity is completely open, but slightly proximal or distal to this region, thin trabeculae partially infill the cavity. The inner and mid-cortex is dominated by CCCB (approx. 21%), which in some sections extends into the outer cortex and may approach the subperiosteal surface (figure 4e–h). Large resorption cavities are concentrated mainly in the mid- to outer cortex (figure 4e,f,i) and are absent from the inner CCCB. Sharpey's fibres are found in some of the bones (figure 4i) and can be seen concentrated along the caudal midshaft of BP/4/1347, consistent with attachment of the adductor and/or quadriceps musculature along the linea aspera region (figure 4g).

**Figure 4.**
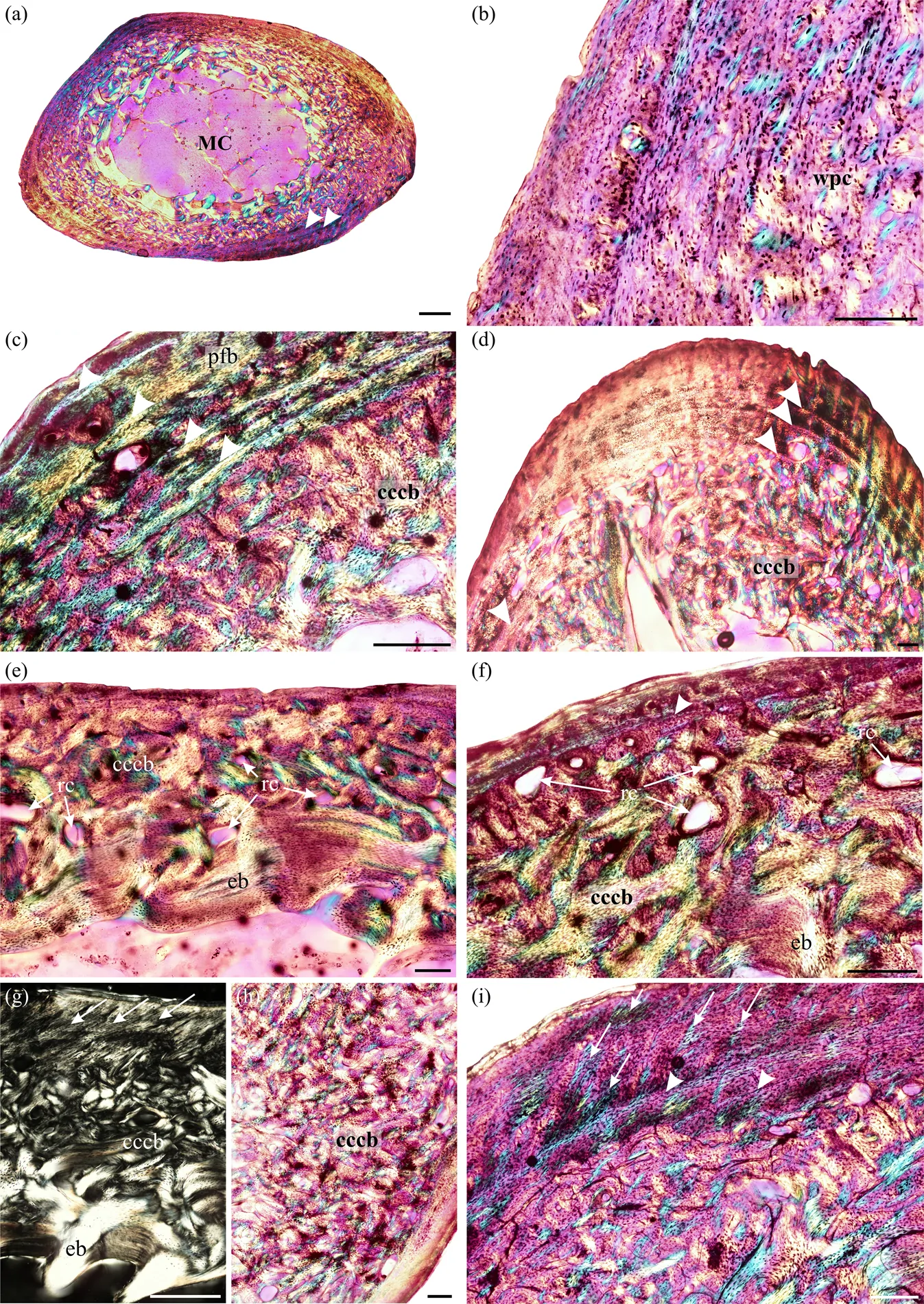
Femoral osteohistology of *Smutsia temminckii*. (a) Whole transverse section of BP/4/1343 showing a relatively thin cortex and large medullary cavity. (b) BP/4/1343 showing a woven–parallel complex. (c) BP/4/1347 showing an outer region of parallel-fibred bone interrupted by four annuli and LAGs. (d) BP/4/1342 showing an inner region of compacted coarse cancellous bone and an outer region of primary bone interrupted by three annuli. (e,f) BP/4/1347 showing thick inner regions of endosteal bone and compacted coarse cancellous bone, and resorption cavities. (g) BP/4/1343 and (h) BP/4/1347 showing compacted coarse cancellous bone almost reaching the subperiosteal surface. (i) BP/4/1343 showing Sharpey's fibres (arrows) indicating muscle insertions. Arrowheads indicate growth marks. Abbreviations: CCCB, compacted coarse cancellous bone; EB, endosteal bone; PFB, parallel-fibred bone; RC, resorption cavity; WPC, woven–parallel complex. Scale bars, 250 µm, apart from (a) = 1000 µm.

### 3.5. Tibia

Four tibiae were analysed. The tibia is a moderately thick bone (CDI = 0.5) with a relatively large open medullary cavity. Similar to the other elements, much of the primary bone tissue has been remodelled with the compact cortex comprising primarily of endosteal bone (13%) and some CCCB (7%) (figure 5a,b). In all sections, endosteal bone lines the medullary cavity becoming thick locally. Where primary bone is visible, in the mid- and outer cortex, a well-vascularized WPC exists with longitudinally oriented primary osteons (figure 5c,d). This bone tissue transitions to poorly vascularized PFB towards the outer cortex (figure 5d), although bone growth was active at the time of death in all individuals. Two LAGs interrupt the primary bone tissue in each tibia (figure 5a). Large resorption cavities are present scattered throughout the CCCB in all the tibiae (figure 5a,e), and secondary osteons are more abundant in the tibiae than in any other element, locally so densely packed as to form dense Haversian bone, comprising approximately 48% of the cortex (table 1 and figure 5f). A localized region of dense Sharpey's fibres is observed in BP/4/1346 on the lateral (interosseous) border.

**Figure 5.**
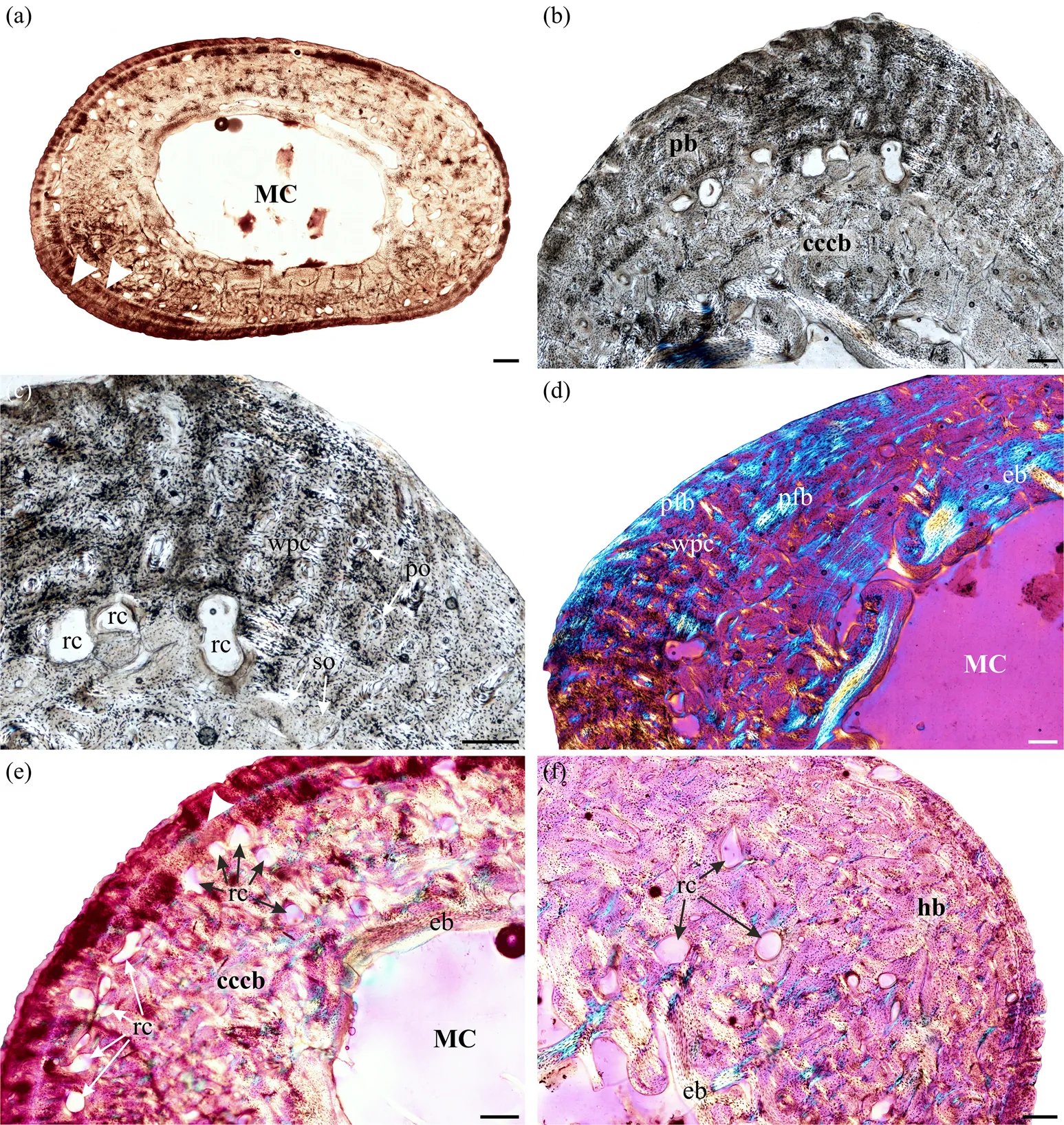
Osteohistology of the tibia of *Smutsia temminckii*. (a) BP/4/1343 showing a moderately thick cortex comprising mostly compacted coarse cancellous bone. (b) BP/4/1346 showing compacted coarse cancellous bone in the inner half of the cortex and primary bone in the outer half of the cortex. (c) BP/4/1346 showing a woven–parallel complex. (d) BP/4/1346 showing a woven–parallel complex and parallel-fibred bone. (e) BP/4/1343 showing large resorption cavities in the compacted coarse cancellous bone. (f) BP/4/1343 showing dense Haversian bone. Arrowheads indicate growth marks. Abbreviations: CCCB, compacted coarse cancellous bone; EB, endosteal bone; HB, Haversian bone; MC, medullary cavity; PB, primary bone; PFB, parallel-fibred bone; RC, resorption cavity; WPC, woven–parallel complex. Scale bars, 250 µm, apart from (a) = 500 µm.

### 3.6. Fibula

Two fibulae, from BP/4/1342 and BP/4/1343, were available for study. The middle of the midshaft is a round bone with a thick compact cortex (Cg = 0.88, CDI = 0.73) and a small open medullary cavity; figure 6a). The bone becomes more triangular in shape proximally. In BP/4/1342, a small region of endosteal bone lines the medullary cavity. The rest of the inner and mid-cortex is secondarily remodelled with abundant secondary osteons (50%, table 1) and CCCB (19% of the cortex) (figure 6a). In BP/4/1343, the CCCB extends to the subperiosteal surface in places and primary bone is only preserved on one side of the outermost cortex, separated from the CCCB by a reversal line. The primary bone consists of a well-vascularized WPC with longitudinally oriented primary osteons (figure 6b,c). A locally thick region of endosteal bone extends from the medullary cavity to the subperiosteal surface in BP/4/1343 (figure 6d). An annulus of lamellar bone traverses the outer primary cortex in BP/4/1342 (figure 6e) and two annuli interrupt the primary cortex in BP/4/1343 (figure 6b,c). Thereafter bone continued to be deposited as poorly vascularized PFB. Resorption cavities excavate areas of the outer cortex in both fibulae. A string of large resorption cavities is observed in sections both from the middle of the midshaft and more proximal ones (figure 6e,f). Secondary osteons are numerous and distributed throughout the cortex.

**Figure 6.**
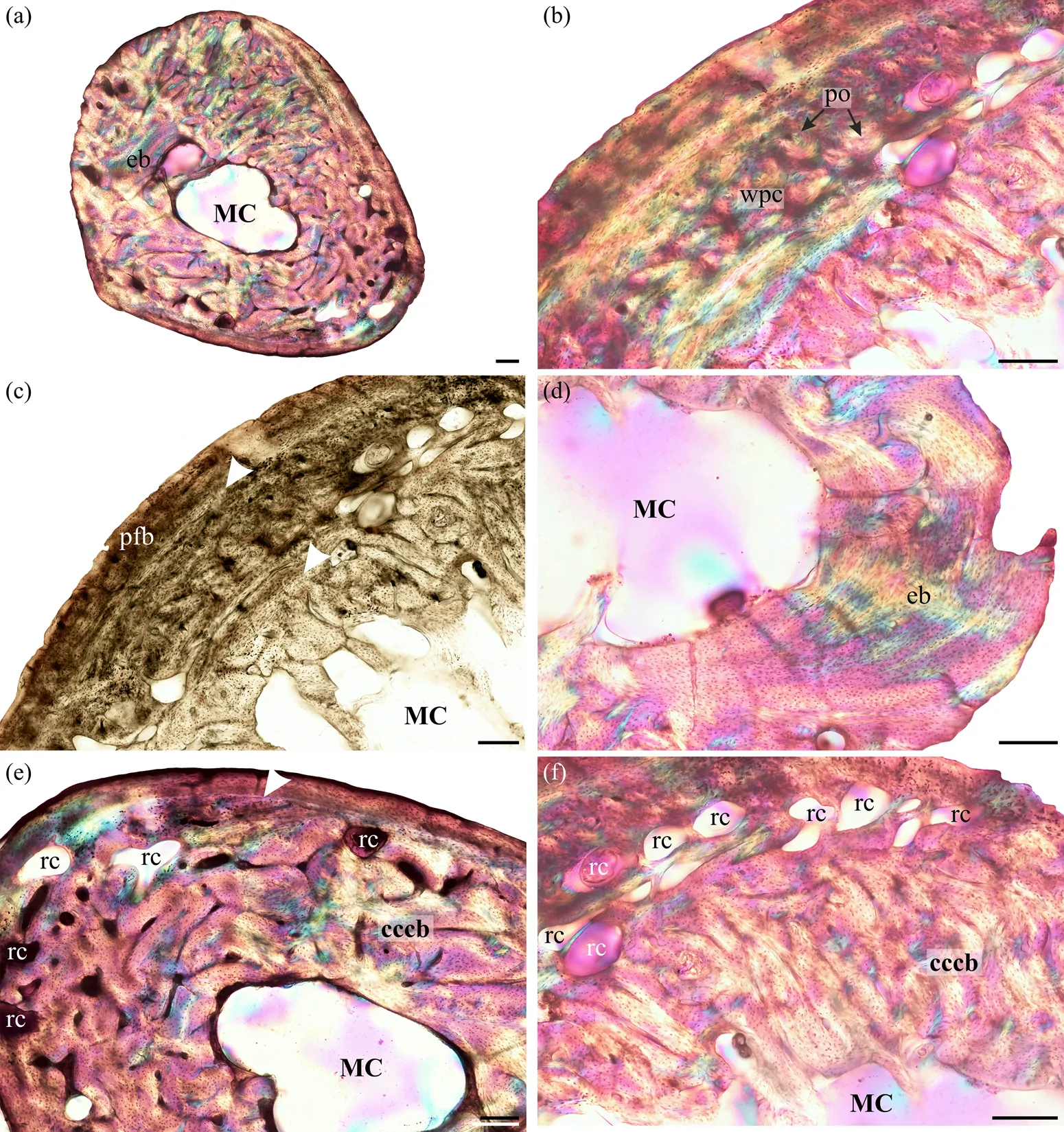
Osteohistology of the fibula of *Smutsia temminckii*. (a) BP/4/1342 showing a thick cortex. (b) BP/4/1343 showing an outer primary cortex of a woven–parallel complex. (c) BP/4/1343 showing a region at the subperiosteal surface of parallel-fibred bone. (d) BP/4/1343 showing a localized thick region of endosteal bone that extends to the subperiosteal surface. (e) BP/4/1342 showing compacted coarse cancellous bone, large resorption cavities and an outer annulus. (f) BP/4/1343 showing an inner region of compacted coarse cancellous bone and a row of large resorption cavities. Arrowheads indicate growth marks. Abbreviations: CCCB, compacted coarse cancellous bone; EB, endosteal bone; MC, medullary cavity; PFB, parallel-fibred bone; PO, primary osteon; RC, resorption cavity; WPC, woven–parallel complex. Scale bars, 250 µm.

## 4. Discussion

Several consistent osteohistological patterns are shared across all appendicular elements of the ground pangolin. In all bones examined, the primary cortex is extensively remodelled, with much of the compact cortex replaced by endosteal bone, CCCB, secondary osteons and large resorption cavities. Where preserved, the primary bone tissue consists of a rapidly deposited WPC with abundant longitudinally oriented primary osteons, which transitions into more organized and poorly vascularized PFB in the outermost cortex. This transition is accompanied by a reduction in vascular density towards the subperiosteal surface and reflects a decrease in growth rate with increasing age. Growth marks, expressed as annuli and/or LAGs, are present in all elements and occur primarily within the middle and outer cortex. Most individuals preserve between one and four growth marks, with BP/4/1342 and BP/4/1347 recording the highest number. However, none of the elements exhibit a distinct OCL, and many sections retain open vascular canals at the subperiosteal surface, indicating that active cortical deposition was ongoing at the time of death. Given that some individuals record at least four annual growth marks, it is noteworthy that they had not reached maximum body size by the time of death and may in fact have been older if earlier growth marks were lost through extensive cortical remodelling. This observation is consistent with the exceptionally low basal metabolic rate (BMR) reported for Temminck's ground pangolin by [21], who estimated a BMR of 123.6 ± 12.6 ml O₂ kg⁻¹ h⁻¹ for a 12 kg individual. Although the woven–parallel complex that characterizes much of the cortical bone indicates relatively rapid osteogenesis, WPC tissues can encompass a broad spectrum of growth rates. In one femur, for example, the growth zones between successive growth marks average only 237 µm in thickness despite the individuals being estimated to weigh approximately 7–8 kg, or only about 60% of the maximum body mass for the species (12 kg). These data suggest that Temminck's ground pangolins require several years to attain maximum size. The osteohistological evidence presented is therefore consistent with the conclusions of [21], who proposed that the slow growth and low metabolic rate may contribute to reduced population recovery following heavy poaching pressure.

A second major pattern is the extensive development of CCCB and endosteal bone, accompanied by widespread secondary remodelling throughout the limb skeleton. All elements show marked cortical remodelling with the most extensive being in the tibia and fibula (figure 7). Abundant resorption cavities are observed in the radius, ulna, femur, tibia and fibula, with many being distributed in the mid- and outer cortex (figure 7). In many sections, rows of partially infilled resorption cavities or secondary osteons form distinct remodelling fronts. Trabecular infilling of the medullary cavity is also common, especially in the humerus and ulna, whereas the femur and tibia generally retain a more open medullary cavity at the midshaft. CCCB is most prevalent in the forelimb bones and the femur. Although extensive cortical remodelling occurs throughout the appendicular skeleton, the tibia (48%) and fibula (50%) exhibit substantially higher proportions of secondary osteons than any other element, indicating particularly extensive intracortical reconstruction in these elements.

**Figure 7.**
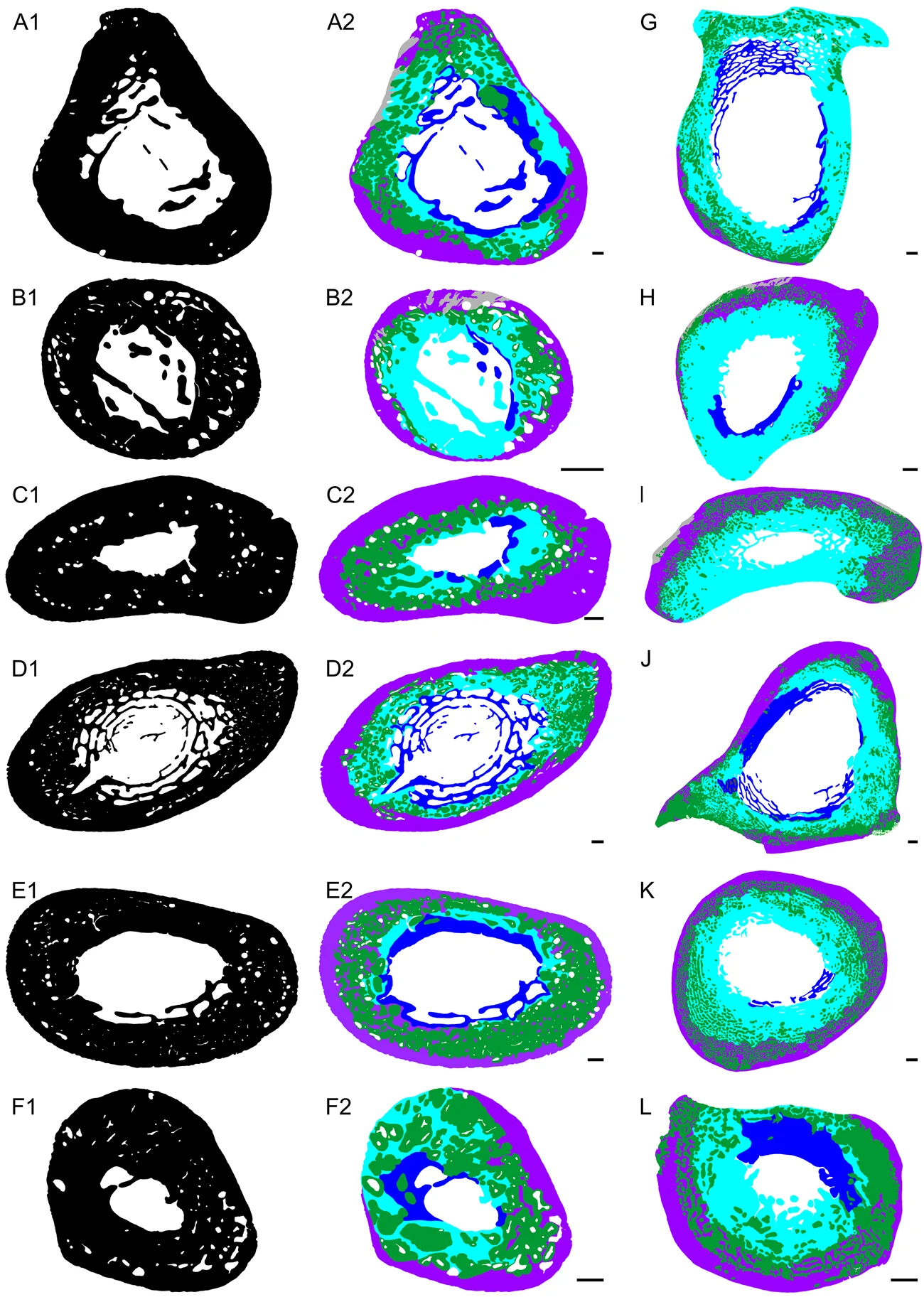
Black and coloured diagrams of the limb bones of the ground pangolin and aardvark. First column, black figures used in bone profiler to obtain bone microanatomy parameters (see text), second column, coloured diagrams of the ground pangolin (A–F) limb bones quantifying different bone tissue types, third column, coloured diagrams of the aardvark (G–L) from [14]. Blue = endosteal bone, light blue = compacted coarse cancellous bone, green = secondary osteons, purple = primary bone, grey = Sharpey's fibres. A1, A2, BP/4/1342 humerus; B1, B2, BP/4/1342 radius; C1, C2, BP/4/1342 ulna; D1, D2, BP/4/1342 femur; E1, E2, BP/4/1342 tibia; F1, F2, BP/4/1342 fibula; G, MVD-M1 humerus; H, MVD-M1 radius; I, MVD-M1 ulna; J, MVD-M195 femur; K, MVD-M1, tibia; L, MVD-M1, fibula. Scale bars, 500 µm for the ground pangolin (A–F) and 1000 µm for the aardvark (G–L).

The distribution of CCCB and secondary osteons suggests that different skeletal elements may respond to mechanical demands through different structural mechanisms. The high density of secondary osteons in the tibia and fibula may reflect cumulative loading associated with weight-bearing, stabilization and force transmission during both locomotion and other aspects of limb use. In the ground pangolin, these demands may be compounded by the crouched posture and robust limb morphology characteristic of the species and by the mechanical requirements of excavation. Similarly, the greater development of CCCB in the forelimb may be consistent with the high mechanical demands placed on the forelimbs during excavation. However, these interpretations should be considered functional hypotheses rather than direct evidence of a fossorial signature, because the relative contributions of locomotion, posture, excavation, body size and other aspects of limb use cannot be distinguished from the present data. The prevalence of secondary osteons (50%), thick cortex (CDI 0.73) and CCCB (19%) in the fibula is particularly noteworthy and indicates substantial cortical remodelling and compaction in this element. Rather than being attributable to a single activity, this combination may reflect the cumulative mechanical demands placed on the hind limb during weight bearing, stabilization, locomotion and excavation.

Sharpey's fibres are another recurring feature throughout the limb bones and are well developed in regions associated with major muscle attachment sites. In the humerus, dense Sharpey's fibres occur along the humeral crest, reflecting strong forelimb musculature associated with digging. The radius preserves Sharpey's fibres near the radial tuberosity and along the shaft margins, likely corresponding to the attachment of the biceps brachii, pronator musculature and interosseous membrane. The ulna also preserves localized Sharpey's fibres associated with extensor and supinator musculature, whereas the tibia and femur show dense fibre development along the interosseous border and linea aspera region, respectively. Overall, the concentration of Sharpey's fibres within the forelimb elements is consistent with enhanced muscular attachment and increased mechanical loading related to scratch-digging and other aspects of limb use.

During ontogeny, CCCB formed in the metaphysis is progressively incorporated into the diaphyseal cortex and subsequently resorbed as the medullary cavity expands, such that CCCB is typically reduced or absent from the mid-diaphysis of mature limb bones [22–24]. The persistence of substantial amounts of CCCB within the midshaft cortex of subadult and adult individuals is therefore unusual. Nevertheless, extensive midshaft CCCB has recently been documented in the aardvark and nine-banded armadillo [14,15]. To facilitate comparison with the ground pangolin, the proportion of CCCB in the limb bones of the aardvark was quantified in the present study (table 1 and figure 7). Aardvarks exhibit substantially greater amounts of CCCB than ground pangolins, with values reaching 59% in the radius and remaining high in the humerus (58%), ulna (47%), tibia (39%), femur (34%) and fibula (26%). The higher proportions of CCCB in the aardvark should, however, be interpreted cautiously. Aardvarks are considerably larger than ground pangolins and are also more specialized excavators, using their powerful forelimbs to actively excavate burrows, whereas ground pangolins are primarily scratch-diggers that modify existing burrows and may also occupy burrows excavated by other animals. Differences in body size, limb morphology and the magnitude and distribution of mechanical loading associated with these contrasting digging behaviours could therefore contribute to the greater development of CCCB in the aardvark, independently of a generalized fossorial adaptation. Although CCCB is most abundant in the forelimbs of both taxa, this pattern does not by itself demonstrate that fossoriality is responsible for its development, particularly given the substantial differences in body size and digging behaviour between the two species.

The distribution of CCCB also differs between the two taxa. In the aardvark, high proportions occur throughout much of the appendicular skeleton, particularly in the forelimb and tibia, whereas in the ground pangolin CCCB is concentrated in the forelimb and femur and is comparatively less developed in the tibia and fibula. These differences become more pronounced for secondary remodelling. The ground pangolin exhibits markedly higher proportions of secondary osteons in the tibia (48%) and fibula (50%) than the aardvark (33% and 38%, respectively). This suggests that the two taxa employ different mechanisms of skeletal reinforcement. Whereas the aardvark appears to strengthen the appendicular skeleton primarily through the retention and compaction of cancellous bone, the ground pangolin relies more heavily on intracortical reconstruction, particularly within the hind-limb zeugopodium. These divergent patterns should therefore not only be read as a direct signature of fossoriality alone. The two species differ not only in the degree and mode of their digging (active burrowing versus scratch-digging) but also in body size, limb morphology, phylogeny and mechanical loading none of which can be separated from fossorial behaviour with our present data. Importantly, Meier *et al.* [25] found no relationship between global humeral bone compactness and degree of fossorial specialization across extant and extinct talpid moles once body size and phylogenetic relationships were accounted for, demonstrating that apparent bone architectural differences between fossorial and less fossorial taxa may reflect other biological variables rather than fossoriality itself. Resolving the relative contributions of body size, mechanical loading, phylogeny and metabolic physiology will require a broader, size-matched comparative dataset spanning fossorial and non-fossorial mammals. Thus, the present comparison supports the interpretation that both species exhibit substantial skeletal reinforcement, but that reinforcement cannot be attributed specifically to fossoriality.

The osteohistological differences between the ground pangolin and aardvark extend beyond the relative abundance of CCCB and secondary osteons. Ground pangolins additionally exhibit abundant, incompletely infilled resorption cavities throughout the middle and outer cortex, a feature that is largely absent in the aardvark and uncommon in most mammals, where open resorption cavities are typically concentrated within the inner cortex [17]. The prevalence and distribution of these cavities suggest that cortical remodelling in the ground pangolin may differ from that documented in other mammals examined to date, including the aardvark, nine-banded armadillo and Cape dune mole rat [13–15]. Although the ground pangolin exhibits extensive secondary remodelling, particularly in the tibia and fibula, many remodelling spaces remain only partially infilled, indicating that intracortical reconstruction was ongoing at the time of death. We hypothesize that this pattern may reflect altered remodelling kinetics, in which osteonal infilling proceeds more slowly than resorption. One potential contributing factor is the exceptionally low basal metabolic rate of *Smutsia temminckii* [21]. Comparative studies suggest that bone turnover is associated with metabolic rate across mammals [26,27], making metabolic physiology a plausible influence on remodelling kinetics in pangolins. However, a direct relationship between metabolic rate and the rate of intracortical remodelling in pangolins has not yet been demonstrated.

Interestingly, extensive CCCB has now been documented in several mammals with relatively low basal metabolic rates, including the aardvark, nine-banded armadillo and ground pangolin, although these taxa differ substantially in body size, phylogeny, ecology and limb function. This recurring association raises the possibility that metabolic physiology may contribute to the persistence or remodelling of CCCB, potentially through effects on bone turnover. However, the present data cannot distinguish the influence of metabolic rate from other potentially important factors, including body size, mechanical loading, phylogenetic history and life-history strategy. Rather than representing a single adaptive response, the persistence of CCCB and the distinctive remodelling pattern observed in *Smutsia temminckii* may reflect the interaction of multiple biological and mechanical factors. Broader comparative sampling of fossorial and non-fossorial mammals, incorporating quantitative measures of body size, limb loading, metabolic rate and phylogeny, will be necessary to evaluate the relative contributions of these factors to CCCB persistence and cortical remodelling.

## 5. Conclusion

Overall, the limb bone histology of the ground pangolin reveals the presence of a woven–parallel complex, which indicates relatively rapid osteogenesis. Multiple growth marks, the absence of an OCL and the retention of open vascular canals demonstrate that individuals remained actively growing for several years and had not attained skeletal maturity even after recording multiple annual growth cycles. At the same time, the widespread occurrence of compacted coarse cancellous bone, extensive secondary remodelling and abundant endosteal infilling indicate substantial mechanical reinforcement throughout the appendicular skeleton. Compared with the aardvark, a larger mammal that actively excavates burrows, ground pangolins exhibit relatively greater secondary remodelling in the hind limb and numerous incompletely infilled resorption cavities extending into the middle and outer cortex. These differences indicate that the two taxa employ different osteohistological patterns of skeletal reinforcement, but these patterns cannot be attributed specifically to fossoriality because the species also differ in body size, phylogeny, limb function and digging behaviour. The prevalence of incompletely infilled resorption cavities in the ground pangolin further suggests that intracortical remodelling may proceed along an unusual trajectory, potentially involving slower osteonal infilling relative to resorption. The exceptionally low basal metabolic rate of *Smutsia temminckii* provides one plausible physiological factor that may influence these remodelling kinetics, although this relationship remains to be tested. Taken together, these findings show that the limb bone histology of the ground pangolin reveals a complex pattern of skeletal growth, reinforcement and remodelling that likely reflects the interaction of mechanical loading, body size, metabolic physiology, phylogenetic history and life-history strategy rather than a single ecological or physiological driver.

## Data Availability

Original renders of histological images are available on MorphoSource [18].
